# Inflammation of the Antrum

**Published:** 1871-02

**Authors:** 


### SELECTED ARTICLES.

### ARTICLE V.

### *Inflammation of the Antrum.*

By Professor Chase.

The antrum or maxillary cavity is of considerable size, and extends on its buccal boundaries from the first bicuspid tooth posteriorly to the posterior roots of the third molar. Its floor is a portion of the palate, and its root the orbit of the eye. Its inner walls forms part also of the cavity, and its outer wall is continuous with the outer alveolar plate of the upper molar and bicuspid teeth. Its only opening is into the nasal cavity, and is a narrow and long one near the top of the maxillary sinus.

The antrum is lined with mucous membrane of the same character as the nasal passages with which it is continuous through the opening just spoken of, and which opening is very much reduced in size when this membrane is inflamed and swollen.

The mucous membrane of the antrum completely lines this cavity, and performs the same function as the mucous membrane of the nose. Inflammation here is so different in character from that of the nasal membrane, and is often concomitant with it.

The roots of the molar and bicuspid teeth often penetrate the antral cavity in their growth, their ends of course being covered with their percimentum, and the mucous membrane of the sinus.

When the teeth thus situated become diseased, they are a prolific source of antral inflammations.

For instance, the pulp of a tooth dies, it disintegrates in its own cavity, the gases produced from its decomposition or putrefaction, pass through the foramini of the roots and create irritation and inflammation in the surrounding pericemental tissue. These roots penetrate the antrum and set up inflammation in its lining membrane. The first symptom of antral disease is a dull pain in that side of the face, and a dryness of the nostril of the affected side.

Now, you must know that in health the lining of the antrum assists by its natural secretion of mucus in keeping the nostril moist. So too, when it participates with the *nose* in inflammation produced by a "cold in the head," it furnishes its proportion of mucus and watery exudation which is so annoying in acute nasal catarrh.

The second antral symptom is this increased flow of mucus, which is the second stage of inflammation in mucous membranes everywhere.

You will be careful then, to examine the nostril of the affected side, in your diagnosis of this disease, so as to discover whether or not the nostril of one side corresponds in condition with that of the other.

Now you must not fail to understand that the opening from the antrum into the nostril may become so closed by inflammation and consequent swelling, that no fluid can pass through it. In this case then the fluid must remain in the antral cavity, as there is no other outlet. If this is really the condition of things, the contents will probably become purulent and there will be considerable pain in the face, rednesss of the cheek, and more or less swelling of the soft tissues.

If science is not called to relieve the patient at this stage, then the bones gradually become absorbed, and growing thin bulge out, either on the buccal or palatine wall, or the orbital. Not unfrequently has the eye been pushed from its socket by this engorgement of the antrum. The osseous wall at last gives way at some point, and the pent-up fluid finds its way through the soft tissues to the surface, forming a fistulous canal and opening at almost any point on the face, even on the under jaw, so as to give rise in some cases to a supposition that one has to do with an alveolar abscess of that bone.

You must not suppose that this is the most frequent way in which antral inflammations progress and terminate. The antro-nasal opening is not so often closed, and then the muco-purulent secretion passes through the nostril, and is always to be found there in greater or less quantity. The best time to see it is after the patient has been lying down, with the head resting on the unaffected side. This position allows the fluid to run out of the cavity into the nostril.

In healthy constitutions this state of things may continue for months and even years without any general disturbance of the system, or without exceeding its present bounds. And it is often unappreciated, the patient supposing that he has only a nasal catarrh. This is more likely to be the case when the exudation passes into the posterior nares and drips into the fauces, instead of appearing at the nostrils.

The exudation in old cases, and repeatedly in bad constitutions is likely to become very offensive. The stench is sometimes intolerable; these cases are likely to be called ozena.

In syphilitic, scrofulous, and scorbutic constitutions, antral inflammation is likely to be chronic and is of the most offensive character.

I have already said that acute nasal catarrh is often the companion of antral inflammation; when the latter is dependent on the former, it is likely to cease when the cure of nasal catarrh is effected.

TREATMENT.

This must be on general principles. Remedies should always be addressed to the general constitution of the patient, and also to that particular phase in which the constition may be at the time.

For acute and antral catarrh you may soak the feet in hot water to the knees. Give a night sweat; hot bricks to the feet and plenty of cold water in the stomach—five or six tumblers full. Blue mass pills of three grains each, at bed time, and a saline cathartic in the morning, may be useful. But my favorite treatment would be the third attenuation of aconite in the inflammatory stage, for half a day, and, if necessary, followed by mercurius vivus third dec. trit., two grains every hour until better. I have often relieved these cases with one dose of the mercury, and effected a cure in from three to four.

You should always examine the teeth of the affected side, and very carefully too, for the cause may lie in an apparently sound one. Press the tooth on examination, from side to side; rap upon it with an excavator handle; look between the teeth for a cavity of decay.

If you find the offending tooth, it will usually respond by pain—a sore feeling more or less extensive. But it may be there will be no pain, only a very considerable looseness of the organ.

It is surprising how the system comes to tolerate a dead tooth, which is causing a constant exudation of purulent fluid into the antrum for months, so well that no pressure upon the tooth produces pain, and yet it may have produced so much absorption in the surrounding tissues that it is loose enough to be extracted by the fingers. No hesitation should be used in *extraction* of such a member, which done, there will probably follow a gush of purulent fluid.

Still you must not surely expect this to be the case, for there may be no opening from its alveolus to the interior of the antral lining mucous membrane, until you push an instrument through it.

Now then, take a crescent drill and explore the bottom of the socket; the bone may need to be drilled away to enlarge the alveolar opening into the antrum, but you may find only soft tissue at the bottom, instead of bony wall. Push through it and explore the antral cavity. Instead of finding an open cavity here, you may find it to be more or less filled with a spongy mass. This is to be torn to pieces in every direction, if present, so that your cautery may act on all its parts where you introduce it.

Whether the cavity is thus filled or free—and it is more likely to be the latter than the former—you will syringe it out thoroughly; first with warm water, then soap and water. If the opening into the nose is patent, you will find much of the fluid injected escaping in that way. After this cleansing, throw into the cavity ten drops of kreosote in an ounce of tepid water. While using the kreosote you will protect the mouth from the regurgitation of the fluid through the alveolus; to this end you can pack some cotton or lint closely around the tube of the syringe, and when the latter is withdrawn, but little fluid will follow it.

To avoid its escaping into the nostrils, let the head be thrown towards the side upon which the extracted tooth was situated. This will bring the opening uppermost, and the cup will not so easily run over.

Instead of kreosote, tincture of iodine or chlorate of zinc may be used, but I prefer kreosote. Carbolic acid may be substituted for the latter, as there is but little difference between them.

A piece of tape should be crowded into the place loosely, to prevent its closing up too soon, which it is not likely to do if you see your patient the following day. You may expect some considerable pain to follow your operations, which will be increased the following day.

You had better *not* repeat the injections as soon as the following day, there is always danger of over-medication. Wait three or four days and observe the result of what you have already done. It may not be necessary to give the cavity a second cauterizing injection.

I have already said that attention should be paid to the general system, and your remedies must correlate with the present dyscrasia of the constitution, whether it be syphilitic, scrofulous, anæmic. This paper would be unduly lengthened were I to particularize in regard to these conditions.

A wholesome and generous diet, however, is never at variance with purulent formations in the antrum.

Let us now suppose a difficult condition of things. For instance, there is no diseased tooth which causes this disturbance! There is no decayed tooth on this side of the upper maxilla! What then? Shall we extract a sound tooth for the purpose of reaching the antral cavity? By no means; sound teeth are not thus to be sacrificed, notwithstanding the authority of surgical authors.

We can very readily penetrate the antrum through the thin wall above the roots of the second bicuspid tooth, or above those of the first molar. Take a strong gum lancet and make a V-shaped cut through the soft tissues on the place designated—the point of the V pointing downwards; then with a trocar or dental drill of large size, bore through the bone. You will not find it very thick—only from an eighth to the thirty-second part of an inch in thickness. Here you can institute your treatment as already directed, being careful to keep the opening patulous, by the use of a strip of tape. I recommend the use of tape instead of lint or cotton wool, for the reason that there is less danger of its getting lost in the antrum, such things have happened, and are not pleasant as they complicate the case sometimes.

If tape should be lost, it can easily be withdrawn by getting hold of one end with an excavator or tweezers. A lint of cotton or wool might be only withdrawn piecemeal as it readily separates.

When this disease has been prodnced by foreign bodies in the cavity, as a dead root which has been forced into it in the operation of extraction, then the socket through which it was drawn is to be enlarged at the bottom with a large drill or burr, and exploration made from this point.

Don't be afraid of making too large an opening; there is no danger but what nature will supply with new bone as large a cavity as you choose to make. One large enough for the root to make its own exit from, easily, is the best.

In all these cases you will find mercnry useful in the manner previously described.

I will now detail a few cases occurring in my own practice, illustrating treatment, and which may be more interesting to you than general descriptions:

Case 1. Mr. B., age thirty, has had the second bicuspid of the left side extracted, thinking it diseased, as he had suffered much pain in that locality. The patient says the tooth extracted was not decayed. Pain continuing, he consults me. There are no decayed teeth on that side of the mouth. None feel sore by rapping them. There is, however, pain on pressing hard against the antral wall. There is, also, unusual dryness of the nasal passage of that side. I diagnose acute inflammation of the antrum. I drill through the vacant socket of the tooth extracted very readily, and syringe with warm water. There was no exudation of any character, when the drill was withdrawn.

Tincture of nut-galls with twenty drops of laudanum is now injected into the antrum, and a tent left in the opening until the next day, when I see the patient, who thinks he is considerably improved. No medication to-day, but cavity is syringed with warm water and tent left out.

Patient called again the fourth day improved; syringed with water and injection of tincture of nut-galls and laudanum. Sixth day patient discharged well.

Case 2. Mr. S., age forty, works in a flouring mill. Sent for me to extract the right upper second molar. I found it undecayed but tender on percussion. I examine right nostril and find some mucus in it, much more than on the opposite side. On enquiry, learn that there is much more in the morning after lying on his left side. Discharge not offensive to smell.

*History.*—Two nights ago, he worked all night in the mill, but took a nap, laying his head upon a bag of damp bran. He took a cold, too, as well as a nap, an got rheumatic pains in his limbs. It seems also that he got rheumatism in the tooth spoken of, that is, pericementitis, or periostitis. This, too, is the way he got inflammation of the antral mucus membrane. The roots of this molar undoubtedly penetrated the antrum.

*Treatment.*— Feet in hot water; go to bed with hot brick to the feet. Mercurius vivus, third decimal trituration, two grains every hour until better. Saw patient next day so much better that medicine was discontinued and patient discharged.

Case 3. Mrs. P., age thirty, scrofulous habit. Present condition: considerable fever, left cheek swollen so as to nearly shut the eye, skin of the face inflamed and red. Left nasal passage obstructed so as to be unable to breathe through it. Slight constant discharge from left nostril of a purulent fluid, greenish color and very offensive. Left bicuspid and molar teeth covered with tartar so as to almost conceal them in the mass. Formerly had tooth ache a good deal on this side.

*Diagnosis.*—Inflammation of antrum from purulent periostitis, following death of a dead tooth which I will find under a mass of tartar. Probable total closure of the antro-nasal opening from chronic irflammation.

*Treatment.*—Removed tartar from the teeth. Find the first molar with crown cavity of decay extending to pulp, which has apparently long been dead. Extract the tooth, a gush of offensive purulent matter flows from the open socket. A large fungus mass covers the ends of all the roots. It is white, and is only a tumor of pericemental tissue. An opening almost as large as the crown of the tooth is found leading from the alveolus to the antrum. This cavity is syringed with soap suds, followed by kreosote—ten drops to an ounce of water.

Thursday. Considerable pain in the cavity yesterday, better to-day, and scarcely any discharge; from the nose it has ceased. Can breathe through the left nostril freely. Injection of tanic acid and water. Fourth day, discharged cured.

Here was an alveolar abscess absorbing floor of antrum and filling cavity with purulent fluids which could be evacuated only by the nose.

Case 4. Mrs. G., washerwoman, age fifty; had some roots extracted from socket of left second molar, at a hospital. Intemperate habits. Syphilitic blood dyscrasia. Has had pain in face ever since the operation, three days since. Muco-purulent discharge from the socket now. Instrument readily finds its way from the socket of the palatine root into the antrum. She does not know whether this root was extracted or not. I suspect that it was forced into the sinus by the root forceps. The floor of sinus probably weakened by alveolar abscess which she says she had there.

The alveolar-antral foraman is too small for explanation. I enlarge it with a rose drill of the largest size, and small chisels. Syringe the parts. Can now explore cavity freely but find nothing. Fill cavity full of warm water and direct the patient to keep the head level, hoping as the water runs out through the alveolus it may bring root to opening. After three or four trials, am rewarded with success. The root comes out with the rush of water, not more than three-sixteenths of an inch long. It has some loose periosteal tissue about the end. Kreosote, five drops to an ounce of water, for injection. No other treatment was found necessary.—*Missouri Dental Journal.*

